# Effects of Chinese Herbal Compound “*Xuemai Ning*”on Rabbit Atherosclerosis Model and Expression of ABCA1

**Published:** 2013-09

**Authors:** Min Chen

**Affiliations:** Department of Geriatrics, Affiliated Hospital of Liaoning University of Traditional Chinese Medicine, Shenyang 110032, Liaoning Province, China

**Keywords:** Atherosclerosis, ATP binding cassette transporter A1 (ABCA1), Blood lipid, Chinese herbal medicine, Reverse Cholesterol Transport (RCT)

## Abstract

**Objective:**

To observe the lipid and the pathological changes of carotid artery smooth muscle cells in atherosclerotic rabbits, verification of Chinese herbal compound which has improve blood lipid and anti atherosclerosis effects, focus on ABCA1 as the key receptor which participated in reverse cholesterol transport, to study the mechanism of Chinese herbal compound (*Xuemai Ning*).

**Materials and methods:**

30 rabbits were randomly divided into blank group, model group and Chinese herbal compound (*Xuemai Ning*) group, The model group and the *Xuemai Ning* group with high fat diet and injection of vitamin D3, causing atherosclerosis model 4 weeks after the intervention of traditional Chinese medicine group, In the 4th week after *Xuemai Ning* group received the intervention of Chinese herbal compound. Blood lipid, the carotid artery pathological changes and expression of ABCA1 gene and protein in peritoneal macrophage surface were detected after 8 weeks.

**Results:**

The carotid artery atherosclerotic plaque formation of the model group was obvious, the carotid atherosclerotic changes of the *Xuemai Ning* group rabbit significantly lighter than the model group. The serum lipid of model group and *Xuemai Ning* group were higher than that of the blank group; and the traditional Chinese medicine can up the expression of ABCA1 protein, higher than those in the model group. Expression of macrophage ABCA1 in model group was significantly up regulated at protein level higher than the blank group; and the traditional Chinese medicine can up regulate the expression of ABCA1 protein, higher than those in the model group. Expression of ABCA1 mRNA was significantly up regulated in model group, ABCA1 mRNA of *Xuemai Ning* group raised more significantly.

**Conclusion:**

*Xuemai Ning* can reduce triglyceride, total cholesterol and low density lipoprotein of hyperlipidemia model in rabbits serum, increase high density lipoprotein, remove foam cells in atherosclerotic cells, improve pathological of AS and up-regulate ABCA1 gene and protein so as to effectively inhibit atherosclerotic disease.

## INTRODUCTION

A large number of studies have demonstrated that hyperlipidemia is the risk factor for stroke, coronary heart disease, myocardial infarction, and sudden cardiac death. Lipid lowering therapy can reverse the atherosclerosis, significantly reduced mortality and related events induced cardiovascular disease. Find natural products with lipid-lowering effect from medicinal plants, has become a research hotspot in recent years (1). The efficacy of Chinese medicine herbal rooted in clinical, with the development of intensive research on cholesterol medicine, it is found and confirmed that Chinese medicine not only has many ways of regulating lipid and intervention effect of multiple targets, but also has definite curative effect and less side effect advantage (2).

Through clinical observation before, we found that the traditional Chinese medicine compound *Xuemai Ning* (composed of Radix Astragali 20 g, Gynostemma pentaphyllum 20 g, hawthorn 25 g, Poria 15 g, salvia15 g, Chuanxiong 10 g, Alisma 10 g, orange peel 10 g, Zhuru 10 g, pseudo-ginseng 3 g, dangshen 15 g), can improve the old patients with dyslipidemia. Previous experimental studies indicated that *Xuemai Ning* could reduce levels of cholesterol, low density lipoprotein and triglyceride of hyperlipidemia rat, increase level of high density lipoprotein (3), ameliorate the expression level of lipoprotein receptor SR-BI, LDLR and CD36, which has the effect of anti AS (4). A lot of researches have proved that effect of HDL anti AS was achieved through reverse cholesterol transport (RCT). ATP binding cassette rotor A1 (ATP-Binding Cassette Transporter A1, ABCA1) mediated RCT is the initial step in cholesterol efflux in flash, which play an important role in the process of RCT. To observe effects of *Xuemai Ning* on RCT and expressions of ABCA1 protein and gene, and explore the possible mechanism of *Xuemai Ning* lipid-lowering effects further.

## EXPERIMENTAL MATERIALS

### Experimental animals

A total number of 30 healthy male( SPF level) New Zealand white rabbits, aged 6-8 weeks and weighing about 2.5 (Kg), were provided by the Laboratory Animal Center of Dalian Medical University [batch number: SCXK (LIAO) 2008-0002].

### Experimental chemicals and reagents

Chinese herbal compound *Xuemai Ning* (ingredients such as before) was a mixture of the single traditional Chinese medicine granules with different doses which provided by Jiangyin Tianjiang Pharmaceutical Co. Ltd. (product batch number 912118).

### High fat diet

2% cholesterol, 10% lard, 0.2% propylthiouracil, 0.5% sodium cholate, 87.3% basic feed.

ABCA1 antibody (Goat anti rabbit): Wuhan boster Biological Engineering Co., Ltd., two anti (mouse anti goat): Beijing Boosen Biological Technology Co. Ltd.

Immunohistochemical SP kit, DAB Kit: Beijing Boosen Biological Technology Co., Ltd.

Trizol, first strand cDNA synthesis Kit: Takara Da lian Company.

The molecular weight of DNA standard agarose, marker: Beijing Iptonic Technologies Co., Ltd.

Gene finder: Shenyang boermei Biological Technology Co., Ltd.

Primers ABCA1 gene in rabbits: Beijing San Bo Polygala Biological Technology Co., Ltd.

We got the ID of ABCA1 gene of rabbit in gene bank, which is 100356765,and part of the mRNA fragment length 187 bp, Therefore, we designed the primer sequences: the upstream5’-GGGGAAGACCACCACCAT-3;Downstream,3’-TAGACCAAGATGCGGGCG-5’. Amplified fragment length 187bp. Rabbit reference gene of β -actin primer sequences for: 5’upstream, -ACCACAGTCCATGCCATCAC -3’; ‘-TCCACCACCCTGTTGCTGTA -3’ downstream, 5. Amplified fragment length 410 bp.

## EXPERIMENTAL METHODS

### Animal grouping and model replication

30 rabbits were randomly divided into the blank group, the model group, and the Chinese herbal group. Rabbits of the blank group were given normal feed, and intraperitoneal injection of physiological saline 2 ml on the first day. The remaining 3 groups fed with high fat diet for 8 weeks, and intraperitoneal injection of vitamin D3 600000 IU/kg on the first day.

### Method of administration

The Chinese medicine group was orally administered Chinese herbal compound (according to the adult normal dosage conversion married rabbit amount) on the fifth week. The model group and blank group was given normal saline, continuous 4 weeks until the end of the experiment.

### Venous serum

Fasting in each group 12 hours before the end of experiment, the whole blood was collected by ear vein for 4 ml on the next day, coagulation promoting tubes, static 1 h, 2500 rpm, centrifugal 10 minutes, collecting supernatant, stored in freezing tube, refrigerator frozen at 80 degrees below zero.

### Peritoneal macrophage

Rabbits of all groups intraperitoneal injection with sterile starch 2 ml 3 days before the end of the experiment, after the end of the experiment collected blood 4 ml from the ear vein to promote coagulation tubes, centrifuge for 10 (2,500 rpm), the supernatant refrigerator at 80 degrees below zero, then both the Auricular vein 10% chloral hydrate anesthesia, Intraperitoneal injection in PBS buffer to 40 ml, Light rubbing abdomen sterile collected in 3 minutes in the peritoneal fluid in the tubes centrifuge 5 min (800 cycles/min), Discard the supernatant and resuspend in DMEM medium high glucose, Counting cells concentrations of 1 × 10^5^/ml and inoculated to cell culture plate 6 hole, 1 ml/hole. Incubation 2 h at 37 degrees, discard the upper suspension cells, remaining the adherent cells of the macrophage. Peritoneal fluid required to exert the maximum amount collected, after incubation each peritoneal fluid of rabbits to keep only two holes, where macrophages attached at the end of culture plate, do not have to collect, as a lipid-loaded macrophages and ABCA1 protein expression samples. Peritoneal fluid of the remaining holes in the Cryopreservation of rabbit sources collected in tubes. Save -80 degrees Celsius, for detection of ABCA1 mRNA expression.

### Carotid artery

Open neck and carotid of rabbits, following take the aorta in 1.5~2 cm and fixed with 4% poly formaldehyde solution, then regular embedding and slice.

### Blood lipid detection

Total cholesterol(TC)（Beijing Jiu Qiang technology Limited by Share Ltd: 13-0128P; TRIGLYCERIDES(TG) (Beijing Jiu Qiang technology Limited by Share Ltd: 13-0114P; high density lipid cholesterol (HDL-C) test kit (chemistry modify enzyme method (KYOWA MEDEXCO., LTD): 380ABG; low density lipid cholesterol (LDL-C) test kit (selective melt method) (KYOWA MEDEXCO., LTD): 585ABH. Instrument: Automatic analyzer (HITACHIC 7600-020).

### Carotid HE staining

The fixed rabbit carotid artery tissue was routine dehydrated, embedded, sectioned and dewaxed, then hematoxylin eosin staining. The carotid artery pathological changes of rabbit were observed under microscope.

### Determination of cholesterol in macrophages

Collected cells on macrophage for detection of lipid loaded amount by oil red O staining, after staining cells were observed under inverted microscope magnified 400 times lipid loaded macrophages and photographs. The macrophages which have been stained by the staining were eluented by 300 ul isopropanol solution of 4%NP-40 after observation, following the eluate was collected on the micro plate and determined by the ELISA instrument under the 492 nm absorbance value (OD492 value).

### The expression of ABCA1 protein was detected by immunocytochemical method

The peritoneal macrophages of rabbits were Collected and dropped on a slide, following incubated at 37°C for 20 min for slide, and then fixed in 2% paraformaldehyde for cell slide. The expression level of ABCA1 in Slide cells were detected by SABC.

### The expression of ABCA1 MRNA in macrophage was detected by RT-PCR

Peritoneal macrophages were collected, joined the trizol 1 ml in the ice, total RNA was extracted from the cells, RNA concentration was determined by UV spectrophotometer, and then reverse transcription for synthesis of CDNA. Following Polymerase chain reaction (PCR) by reverse transcription products, reaction system of 50 ul, the annealing temperature was set to 65°C and extending temperature was set to72°C then the amplification products in agarose gel of 1.5% for electrophoresis, while the image acquisition and the determination of integral optical density value were showed in gel analysis system, and analysis of data.

### Statistical methods

Date was subjected to analysis using the Statistical package for social sciences (spss), version 10.00. The measurement data with the mean standard deviation (X ± s), multi group were compared with single factor analysis of variance, *P*<0.05 was statistically significant.

## RESULTS

### The pathological changes of carotid artery

**Figure 1:**
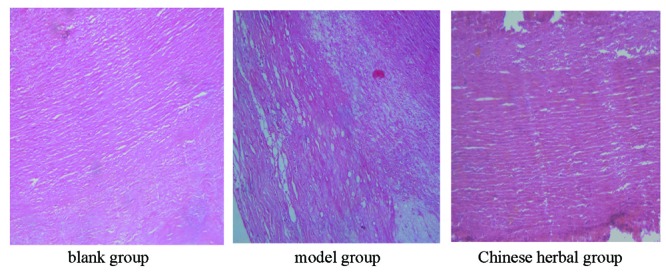
Pathological changes of All rabbits carotid artery (HE ×100 times).

We observed the pathological changes of carotid artery by HE staining, carotid artery vascular smooth muscle cells of the blank group were very regular line-up,bright color, there were no lipid deposition, no lipid spots and atherosclerotic plaque. In the model group, carotid artery wall thickening, into the cavity was outstanding; there were a lot of sub initial lipid depositions, large number of foam cells were visible and obvious atherosclerotic plaque. The pathological changes of carotid artery as of the Chinese herbal group was significantly lighter than the model group (Figure 1).

### Determination results of Rabbit blood lipid content

**Figure 3:**
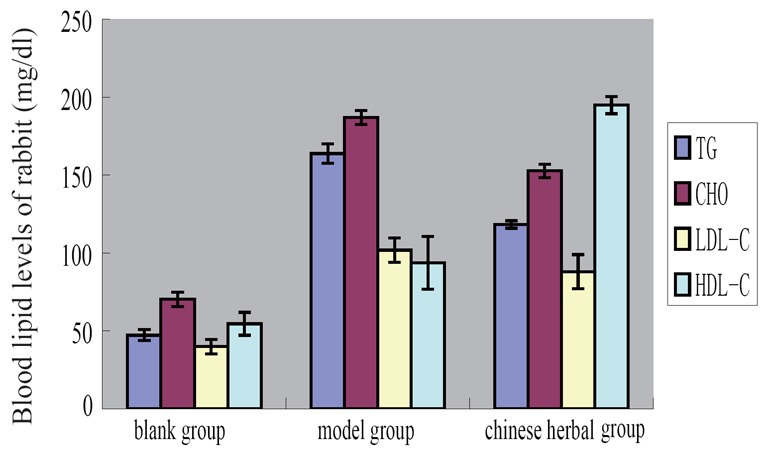
Determination results of Rabbit blood lipid content.

The levels of cholesterol (CHO), triglycerides(TG), high-density lipoprotein cholesterol (HDL-C)and low-density lipoprotein cholesterol (LDL-C) in the rabbit serum of model group and Chinese medicine group were significantly higher than that of blank group (*p<0.01*). The contents of TG, LDL-C in rabbit serum cholesterol of Chinese medicine group is significantly lower than the model group, and HDL-C content is significantly higher than the model group (*p<0.01*) (Table 1, Figure 3).

**Table 1:** Determination results of Rabbit blood lipid content (X ± s, n=10)

Groups	Rabbit blood lipid levels (mg/dl)			
	TG	CHO	LDL-C	HDL-C

Blank group	47.35 ± 3.48^b^	70.33 ± 4.55^b^	40.00 ± 4.57^b^	54.63 ± 7.27^b^
Model group	163.78 ± 6.25^a^	186.94 ± 4.54^a^	101.78 ± 7.90^a^	93.81 ± 16.95^a^
Chinese herbal group	118.32 ± 2.47^a^ ^b^	152.64 ± 4.23^a^ ^b^	88.08 ± 10.88^a^ ^b^	194.88 ± 5.50^a^ ^b^
F	1187.411	1480.630	131.027	473.485
*P*	0.000	0.000	0.000	0.000

a Compared with the blank group, *p*<0.01;

b Compared with model group, *p*<0.01.

### Observation of macrophage-foam cell formation

**Figure 2:**
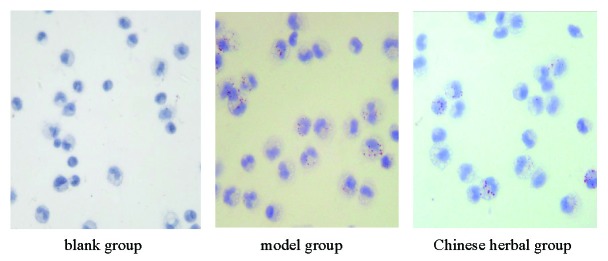
In rabbit peritoneal macrophage lipid-loaded (oil red o stain, ×400 times).

Under the microscope, macrophages are round, volume is larger, the nucleus is round, Reni form, horseshoe and irregular shape, in cytoplasm with numerous red lipid particles, and gathered into droplets, with foam cell morphological characteristics (Figure 2).

### The results of the amount of lipid loaded macrophages

**Figure 4:**
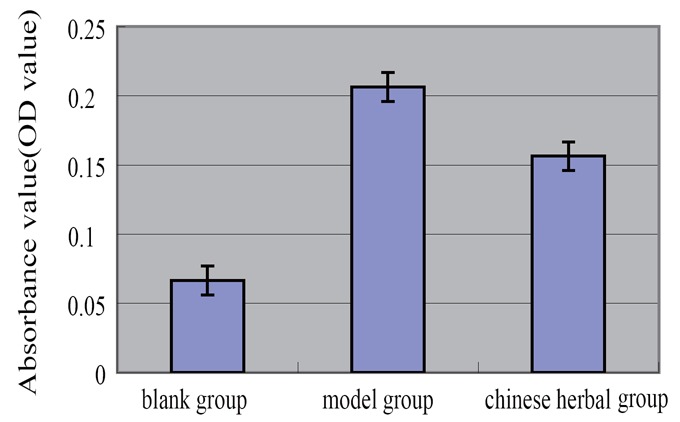
The results of the amount of lipid loaded macrophages.

The amount of lipid loaded in rabbit peritoneal macrophages of model group was significantly more than the blank group and Chinese medicine group. The eluate was determined absorbance value after elution with the isopropanol solution of 4%NP-40, and the absorbance value of model group was significantly higher than the other two groups (Table 2, Figure 4).

**Table 2:** Detection results of peritoneal macrophages cholesterol of rabbit (X ± s, n=10)

Groups	N	Rabbit peritoneal macrophages (relative to the amount of lipid loaded quantity, OD)

Blank group	10	0.0665 ± 0.0063^b^
Model group	10	0.2064 ± 0.0104^a^
Chinese medicine group	10	0.1564 ± 0.0068^a^ ^b^
F		554.947
*P*		0.000

a Compared with the blank group, *p*<0.01;

b Compared with model group, *p*<0.01.

### Expression of ABCA1 protein in macrophages

**Figure 5:**
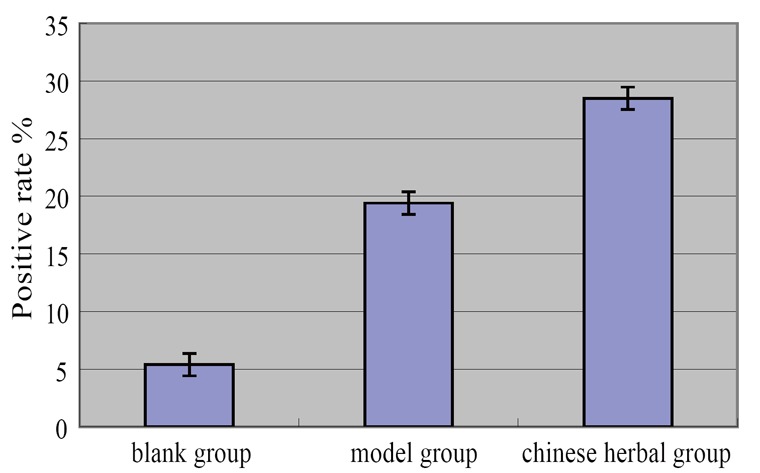
The expression level of ABCA1 protein in peritoneal macrophages.

**Figure 6:**
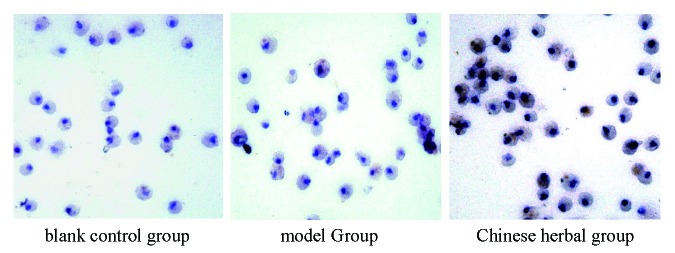
In rabbit peritoneal macrophage cell surface expression of ABCA1 protein (DAB color rendering, ×400 times).

Expression of ABCA1 protein was significantly up-regulate in model group and higher than those in the blank group (*P<0.01*). Statistics of the percentage of positive cells showed that the expression of ABCA1 protein of Chinese medicine group was significantly unregulated compared with model group (*P<0.01*) (Table 3, Figure 5, Figure 6).

**Table 3:** The expression level of ABCA1 protein in peritoneal macrophages (X ± s, n=10)

Groups	N	Expression level of macrophage ABCA1 protein (positive rate %)

Blank group	10	5.40 ± 0.97^b^
Model group	10	19.40 ± 1.65^a^
Chinese medicine group	10	28.50 ± 1.72^a^ ^b^
F		478.542
*P*		0.000

a Compared with the blank group, *p*<0.01;

a Compared with model group, *p*<0.01.

### Rabbit abdominal macrophage ABCA1 mRNA expression results

**Figure 7:**
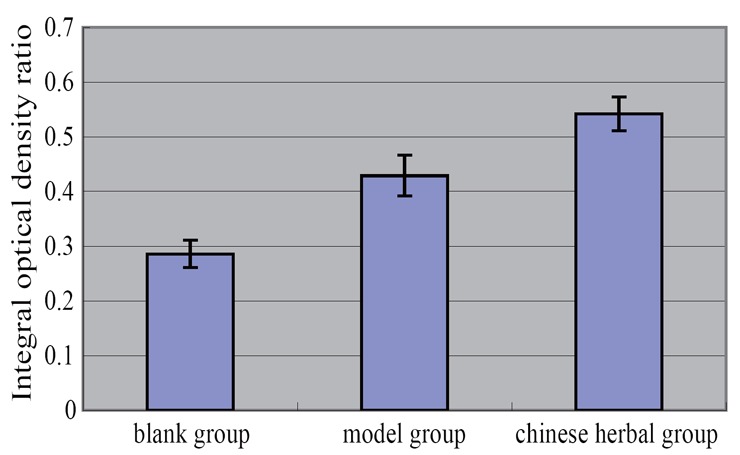
Rabbit abdominal macrophage ABCA1 mRNA expression results.

**Figure 8:**
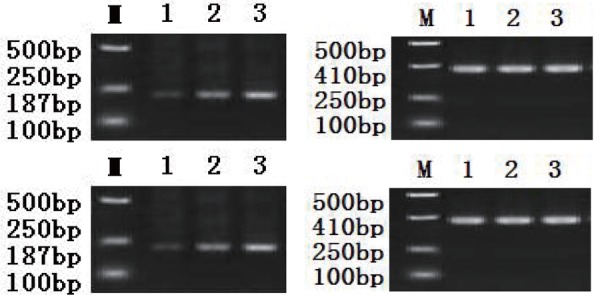
Rabbit abdominal macrophage ABCA1 mRNA expression results. 187 bp ABCA1 gene amplification products; 410 bp beta-actin gene amplification products. M, Marker; 1. blank group; 2. model group; 3. chinese herbal group.

Expression of ABCA1 mRNA of the model group, Chinese herbal group and control group were significantly increased, compared with the control group *P*<0.01. Chinese herbal group and the control group was higher than that in the model group, *P*<0.01 (Table 4, Figure 7, Figure 8).

**Table 4:** Rabbit abdominal macrophage ABCA1 mRNA expression results (X ± s, n=5)

Group	N	The expression level of ABCA1 mRNA in rabbit peritoneal macrophages (integral optical density ratio)

Blank group	5	0.286 ± 0.025^b^
Model group	5	0.429 ± 0.037^a^
Chinese medicine group	5	0.542 ± 0.031^a^ ^b^
F		58.964
*P*		0.000

a Compared with the blank group, *p*<0.01;

b Compared with model group, *p*<0.01.

## DISCUSSION

New Zealand rabbit has long been considered the best choice to establish hyperlipidemia and AS model, because the blood lipid spectrum is very similar to human (5). Exogenous cholesterol absorption rate as high as 75~90% in rabbits, If the pure cholesterol dissolved in vegetable oil fed rabbit, which can cause Hypercholesterolemia Rabbits typical, accompanied by changes in aortic atherosclerotic coronary arteriosclerosis, pathology. Our results shows that Lipid rabbits model higher than those in the blank group, The carotid artery wall thickening, into the cavity is outstanding, a lot of sub initial lipid deposition, large number of foam cells were visible and obvious atherosclerotic plaque, We successfully established the model of atherosclerosis.

We observed in the previous experiment that *Xuemai Ning* can reduce levels of high fat serum cholesterol, low density lipoprotein and triglyceride of rat, the effect of *Xuemai Ning* and atorvastatin (10 mg) showed no significant difference in statistics.

Rabbits were used to replicate the model of hyperlipidemia and atherosclerosis successfully in this experiment, results show that Chinese herbal *Xuemai Ning* can reduce content of cholesterol, triglyceride and low density lipoprotein, increase the content of high density lipoprotein, it also can improve the pathological changes of the carotid artery. We further experiments in order to explore the mechanism of *Xuemai Ning*.

Foam cell formation is characterized with early atherosclerosis, research shows that there was oxidized low density lipoprotein receptor in macrophage surface, which can identificate oxidized low density lipoprotein in the serum and ingeste it into cells. Due to the accumulation of lipid, lead to excessive intake of macrophages on low density lipoprotein and foam cell formation.

Our results show that the lipid content of the model group was significantly increased in macrophages.oil red O staining showed strong positive, there were a large number of red lipid droplets in macrophages under the microscope, The micro plate assay lipid loaded macrophages were significantly more than the blank group. After treatment of *Xuemai Ning*, rabbit macrophage cholesterol content decreased. These results suggest that *Xuemai Ning* can scavenge lipid composition of macrophages in the role. However, what is clear on the lipid composition in foam cells by what mechanism? We conduct a thorough study in order to understand the mechanism.

Foam cells which formed by lipid loaded macrophages accumulation are cell marker in atherosclerotic lesions, it is important significance to promote cholesterol efflux from foam cells and prevent the development of atherosclerosis (6, 7). RCT is the most important route of the body expel excess cholesterol and one of the most important body mechanism of anti atherosclerosis according to the currently research (8), RCT refers to the peripheral tissues (including macrophages) free cholesterol transport to the liver by lipoprotein and the hepatic excretion process (9). A series of studies confirm that cholesterol efflux can occur in fibroblasts, adipocytes, macrophages and peripheral tissue (10-12). Comparing with cholesterol efflux of other peripheral tissue, cholesterol efflux of macrophages, although only a very small part of peripheral cholesterol on atherosclerosis, but the most significant. Macrophages are major cellular components of atherosclerotic lesions and form into foam cells, which deposit in the arterial wall formation of early atherosclerotic plaque. Therefore the macrophage RCT plays a key role for the development of AS. At present, promote macrophage RCT become the research direction of anti atherosclerosis HDL (13).

It has become clear that the macrophage cholesterol efflux mainly through 3 ways: 1) ATP binding cassette transporter (ABC) A1 combined with free apolipoprotein (apo) AI or B-HDL; 2) ABCG1 combined with the mature HDL; and 3) scavenger receptor class B type I (SR-BI) combined with the mature HDL, which plays a key role on macrophage cholesterol efflux. Therefore, the process of RCT in macrophages were mediated by ABCA1, ABCG1, and (SR-BI) mediated, or more comprehensive pathway.

ABCA1 belongs to the ATP binding cassette transporter family (ATP-binding, cassette transporter, ABC), is a kind of important receptor protein of the cell membrane, which take ATP for energy and carry on transmembrane transport of various metabolites, lipids, cholesterol, cell toxins and drugs etc, as an important mechanism of reverse cholesterol transport (14, 15).

Research on Adorni MP and Haghpassand M results show that when the expression of ABCA1 protein of macrophage surface down regulation, cholesterol efflux to poor lipid apoA-I decreased (16, 17). Another study reported that when ABCA1 protein deficiency, the role of macrophage RCT also significantly decreased (18, 19). Van Eck study found that when the over expression of ABCA1 protein of macrophage surface, cholesterol efflux to poor lipid apoA-I significantly increased (20). In normal mice transplanted into ABCA1 knockout mice bone marrow, the mice atherosclerosis injury increased significantly. Over expression of ABCA1 of murine bone marrow in normal mice were transplanted and the injury degree in atherosclerosis decreased (20).

There have been reports of cytokine tumor necrosis factor (TNF)-alpha, interleukin (IL)-1 beta and interferon gamma, unsaturated fatty acid, C reaction protein (21) and angiotensin (22) can down regulate the expression of ABCA1 transcription and protein. While IL-10 can antagonize the effect of TNF-α, promotes the expression of ABCA1 (23); Estrogen, caveolin-1 (24), cyclooxygenase 1, 2 and prostaglandin D2, E2 can also raise the expression of ABCA1.

Following are reports about effects of drugs relate to regulation of lipid in recent study. Rubic *et al* found that nicotinic acid can stimulate up-regulation of monocytes peroxisome proliferator activated receptor gamma (PPAR γ) and expression of ATP binding cassette transporter A1 (ABCA1) (25). It is also reported that nicotinic acid can promote fat cell cholesterol efflux (26) and up regulate the expression of ABCA1 in aorta of hypercholesterolemic rabbits (27). It is reported that Bezafibrate promote macrophage RCT by increasing the expression of ABCA1, ABCG1and SR-BI (28). Anther study showed that Probueol promoted macrophages RCT *in vivo* in mice dose dependently, and Probueol promoted cholesterol efflux from the macrophages possibly had no relation to the expression of ABCA1 and SR-Bl (29).

This results show that the expression of ABCA1 protein and gene in macrophage surface of model group were significantly higher than those in the blank group (*P*<0.01), which indicated that expression of ABCA1 protein and gene in atherosclerotic rabbits macrophage were elevated; Expression of ABCA1 protein and gene in macrophage surface of rabbits in *Xuemai Ning* group was significantly increased than in model group (*P*<0.01), which indicated that *Xuemai Ning* further increase expression of ABCA1 protein and genein the macrophage surface. Our analysis is that when the body accumulated a large number of lipid composition and lipid excessive intake of macrophage form into foam cells, then activate the body’s self protection and raise the expression of ABCA1 through certain channels, so as to assist in macrophages to reverse transport of cholesterol and keep clear of *in vivo* lipid composition. However, when the lipid composition accumulated too much and beyond the compensatory ability of the body, fail to prevent forming foam cell finally. While *Xuemai Ning* can up the expression of ABCA1 protein and gene and promote to remove lipid content in foam cells effectively might be one of the mechanisms to improve the atherosclerotic pathological changes.

*Xuemai Ning* which composed of natural herbal, has effects of regulating dyslipidemia and anti-atherosclerosis, however, the effective components of *Xuemai Ning* need in future experiments to further clarify. RCT is a complex process, involving a variety of proteins, receptors and transporters, effects of lipid-lowering drugs currently on RCT need to be studied, we will do in-depth study of *Xuemai Ning* on RCT and make control research of the lipid regulating drug in the next step of the study.

## CONCLUSION

1. *Xuemai Ning* can effectively reduce content of serum triglyceride, total cholesterol and low density lipoprotein of hyperlipidemia and atherosclerosis model in rabbits, raise level of high density lipoprotein;
2. *Xuemai Ning* can effectively improve the pathological changes of rabbit abdominal aorta atherosclerosis;
3. *Xuemai Ning* can effectively remove the lipid content in foam cells;
4. *Xuemai Ning* can up regulate the expression of ABCA1 gene and protein;
5. Up-regulating the expression of ABCA1 gene and protein and eliminating lipid in foam cells may be one of the mechanisms of prevention and treatment of atherosclerosis of *Xuemai Ning*.
